# Psychometric properties of the CEMA-A questionnaire: motives for lying

**DOI:** 10.3389/fpsyg.2023.1289209

**Published:** 2023-12-20

**Authors:** Enrique Armas-Vargas, Rosario J. Marrero, Juan A. Hernández-Cabrera

**Affiliations:** ^1^Department of Clinical Psychology, Psychobiology and Methodology, Universidad de La Laguna, Tenerife, Spain; ^2^Instituto Universitario de Neurociencia (IUNE), Universidad de La Laguna, Tenerife, Spain

**Keywords:** motives for lying, intrapersonal motivation-emotionality, interpersonal motivation-sociability, egoism/hardness motivation, malicious motivation

## Abstract

Previous research on the motives for lying lacks factorial models that allow grouping of motives into specific categories. The objective of this study is to confirm the factorial structure of the questionnaire of motives for lying (CEMA-A). Participants were 1,722 adults residing in the Canary Islands (Spain) who completed the CEMA-A and the Eysenck Personality Questionnaire (EPQ-R). The four-dimensional structure of the questionnaire was confirmed (*χ*^2^ = 1460.97, df = 325, *p* = 0.001; CFI = 0.94; TLI = 0.93; NFI = 0.93; NNFI = 0.93; RMSEA = 0.05, CI = 0.051–0.057; SRMR = 0.04). The four factors of the CEMA-A were Intrapersonal Motivation–Emotionality, Interpersonal Motivation–Sociability, Egoism/Hardness Motivation, and Malicious Motivation, with an internal consistency between 0.79 and 0.91. Invariance analyses confirmed the equivalence of the instrument for men and women. The CEMA-A factors positively correlated with Neuroticism and Psychoticism, and negatively with Dissimulation. Extraversion was not related to any of the factors, and only displayed a low negative correlation with Intrapersonal Motivation–Emotionality. Analysis of variance showed that men scored higher in Egoism/Hardness and Malicious Motivation. The CEMA-A has proven capable of apprehending the motives for lying and has adequate psychometric criteria for use in various populations.

## 1 Introduction

A lie is a multidimensional construct (DePaulo et al., 1996; Phillips et al., 2011; Muzinic et al., 2016), defined as a form of verbal deception, where there is a deliberate attempt to hide, falsify, generate and/or manipulate, in some way, factual, and/or emotional information, to encourage in the other a belief that the communicator themself considers false (Knapp and Comadena, 1979; Ekman, 1985/2001; Miller and Stiff, 1993; Buller et al., 1994; Masip et al., 2004; Vrij, 2008). People evaluate lying from two positions, by assigning a negative image to those who lie and by rationalizing or justifying the lie when it is used by the individual themself (Nyberg, 1993; Kashy and DePaulo, 1996; Bond and DePaulo, 2006). Thus, more intentionality is attributed, and the label of liar is assigned more to others than to oneself when lying (Curtis, 2021). Research suggests that people view their everyday lives as small, and unimportant, rarely plan them, and unconcerned about being discovered (DePaulo et al., 2004; Bond and DePaulo, 2006). Most lies that are considered serious are motivated by the desire to cover up a personal fault, a discredited fact or to hide transgressions that, if discovered, could have serious consequences for the identity and reputation of the liar (McCornack and Levine, 1990; Metts, 1994; DePaulo et al., 2003a, 2004). These types of lies are more carefully planned, and are often unjustifiable, immoral, or illegal (DePaulo et al., 2004). Therefore, unless there is a psychopathological problem (Curtis and Hart, 2022), people often use deception, when telling the truth is a problem (McCornack et al., 2014; Levine et al., 2016; Moshagen et al., 2020). Since lying is intentional, that people lie for a reason or motive is implicit (Bond and DePaulo, 2006; Levine et al., 2010), lying, in itself, is not a goal, but a means to achieve another (Levine et al., 2010). For example, someone tells their partner that they are at home (a lie) when they are in fact with a lover. This lie does not seek to convince the partner of their where about, since they could have excused themselves in another way, to convince them of their fidelity (the goal).

In general, people may tell a lie at some point, despite it being considered a reprehensible act with harmful consequences (Bok, 1978; Teasdale and Kent, 1995; Solomon, 2009; Curtis and Hart, 2015). However, lying every day is not common behavior for most people (Serota et al., 2010, 2022; Serota and Levine, 2015). Studies in the field of everyday lies find that people report an average of one to two lies a day (DePaulo and Kashy, 1998; Serota et al., 2010; Serota and Levine, 2015). However, the average may be distorted by extreme scores from people who often lie. These differences in the frequency of lying may also be related to sociodemographic variables. Some studies suggest that young people and men admit to lying more often (DePaulo et al., 1996; Serota et al., 2010; Armas-Vargas, 2017a), although the difference in frequency of lying between men and women is very small (Gerlach et al., 2019). Other research has found that gender differences vary depending on the subject matter of the lie (DePaulo et al., 1996; Feldman et al., 2002; Haselton et al., 2005; Erat and Gneezy, 2012). Various studies suggest that lying decreases with age (Jensen et al., 2004; Serota et al., 2010). Adolescents tend to lie more than university students, who do so less than the general adult population (DePaulo et al., 1996; Serota et al., 2010; Levine et al., 2013; Armas-Vargas, 2020, 2021b).

Most research has been carried out in the area of lie detection (Vrij and Ganis, 2014). The truth-default theory (Levine, 2014; Levine et al., 2022) is one of the most widely accepted theories about human deception detection. This theory proposes that people tell the truth by default, that is, they are honest most of the time, and are more likely to believe that others tell them the truth rather than lies. Thus, people do not usually lie except when the truth is an obstacle to goal attainment (Levine et al., 2010). However, if a situation becomes problematic, people can then lie. On the other hand, the self-concept maintenance theory (Mazar et al., 2008; Ariely, 2012) proposes that people are more likely to lie when the ego is depleted (Mead et al., 2009). Therefore, the aim of a person who deceives is to satisfy complex intrinsic motivations, such as maintaining a favorable self-concept (Mazar et al., 2008). Similarly, from a self-presentational perspective, DePaulo et al. (2003b) propose that people mainly lie for psychological reasons to protect or to give a better image of themselves, that is, to deliberately try to manage others’ impressions of them. Furthermore, DePaulo et al. (2003b) suggest that deception and truth can be distributed along a continuum rather than considered different dimensions. The reasons for lying or telling the truth are the same: people are interested in giving a good image or describing important aspects of themselves. However, self-presentation is not the only reason why one can lie. In a transcultural study, Levine et al. (2016) found that there were different types of deception motives such as maintaining a positive self-image, protecting others, avoiding others, seeking an advantage, social politeness, hiding a transgression, being malicious, and joking.

Furthermore, some people who lie give socially desirable responses and misrepresent their motivations for lying (DePaulo et al., 2003b). Much research indicates that people offer a positive view of themselves, highlighting positive features such as that they are better, more honest, and more moral than others (Alicke et al., 1995). These beliefs are identified with self-deception. For self-deception to produce positive effects on the person, individuals must, by definition, be unaware of its illusory basis (Baumeister, 1993). According to Trivers (2002), “the hallmark of self-deception in the service of deceit is the denial of deception, the unconscious running of selfish and deceitful ploys, the creation of a public person as an altruist and a person beneffective in the lives of others” (p. 276). Therefore, as they are not fully aware of their motivations for lying, these people can confidently and “honestly” claim that their lies were altruistically motivated. However, according to cognitive dissonance theory (Festinger, 1957), altruistic interpretations of deception may not completely dispel the dissonance of the person lying. In these cases, the person may wield feeling guilty about the lie as a way to reduce cognitive dissonance. When they express guilt for lying to others, they are reinforcing their positive view of themselves. Individuals who feel and express guilt for their misdeeds are often considered better people than those who show no remorse (Baumeister, 1997).

Other authors have attempted to capture and classify the motives for lying. Turner et al. (1975) list five motivations for lying: (a) to save face (to protect identity, self-esteem), (b) to manage or handle relationships (to end a relationship), (c) to exploit others (by manipulating, having control, power, and influence over the other), (d) to avoid tensions or conflicts (controlling a conversation to avoid it being uncomfortable or triggering an argument) and (e) to control situations (to maintain, redirect or end interaction with the other). Buller and Burgoon (1996) point out that lying is employed for three main reasons/motives: (a) “instrumental” (to gain power, influence others, avoid disapproval, or do harm), (b) “identity” (to improve the image we present to others, avoid shame, improve or protect self-esteem, and increase social desirability), and (c) “relational” (to influence our relationships with others).

Another proposed categorization of the motives for lying is based on (a) whether the liar is “centered on themselves” (egotistical, to protect themselves) or on the other person (to protect others), and (b) whether the liar is “altruistically” or “maliciously” motivated (DePaulo et al., 1996, 2003a; DePaulo and Kashy, 1998; Vrij, 2000). Altruistic lies also allow one to protect one’s well-being (Ennis et al., 2008) and have been classified as considerably more acceptable than egotistical lies (for one’s own benefit or for malicious purposes) (Lindskold and Walters, 1983; Seiter et al., 2002). DePaulo et al. (1996) found that people lie far more about themselves than they do about others. The motives behind the lies were mostly selfish, and many more lies were told for emotional reasons (to protect themselves from shame, or their own feelings) than for personal advantage (to obtain benefits or material gain).

From a qualitative perspective, the motivations for lying have been classified from two dimensions: protective versus beneficial lies and self-oriented versus other-oriented lies (Arcimowicz et al., 2015). The combination of both dimensions facilitates the identification of four types of lies: egoistic (self-oriented/beneficial), self-defensive (self-oriented/protective), pleasing (other-oriented/beneficial), and sheltering (other-oriented/protective). Some egoistic lies cited in the interviews were for material gains or admiration from others. Self-defensive lies included avoiding responsibility, discussions, or negative consequences. Pleasing lies were related to making someone happy, and sheltering lies were associated with protecting someone from distress or avoiding hurting someone else. These last two categories were more difficult to distinguish. In general, people lie primarily for protective motivations that allow them to avoid punishment rather than for personal benefits.

The role played by inter-individual differences may affect the probability of lying (McLeod and Genereux, 2008), as well as the different motives for lying and achieving certain goals or desires (Buller and Burgoon, 1996; Olson and Weber, 2004). Some studies point out the importance of personality traits in the probability of and motives for lying (McArthur et al., 2022). Machiavellianism or extraversion are associated with frequency and different types of lying (Kashy and DePaulo, 1996; McLeod and Genereux, 2008; Hart et al., 2019). In the prison population, lying has been found to be associated with both neuroticism and psychoticism (Gudjonsson and Sigurdsson, 2004). Fullam et al. (2009) found that people with a high level of psychoticism showed a low level of conditioning to social norms, a low level of fear and avoidance of harm, and were more likely to lie. The results of the study revealed the importance of analyzing the role of the traits of insensitivity and emotional deficit (typical of psychoticism and neuroticism) in the tasks that evaluate the cognitive elements that may be involved in deceiving and manipulating others. Giammarco et al. (2013) also found an association between greater ability to deceive and the Dark Triad of Personality (Machiavellianism, psychopathy, and narcissism). Furthermore, an important motivator in lying is emotions (Ekman, 1985/2001). Lying is mainly motivated by negative emotions, such as anxiety, fear (Ekman, 1985/2001; Tangney et al., 1996), or guilt, which arises when there is a discrepancy between internalized values and actual behavior (Mosher, 1968; Ekman, 1985/2001; Millar and Tesser, 1988); shame, when a person does not meet their own personal moral standards (Keltner and Buswell, 1996; Tangney et al., 1996; DePaulo et al., 2003a); and insecurity, fear of rejection and criticism (Armas-Vargas, 2021a,b). People are motivated to lie mainly through certain emotional needs, which are satisfied through social interaction, the instrumentalization of relationships, or harming others (Armas-Vargas, 2021a). That is, personal/emotional motives may be based on other more social, instrumental/selfish, or malicious motives (Armas-Vargas, 2021a). Many of these emotional motives may be implicit or escape awareness (McClelland et al., 1989; Bargh, 1990; Bargh and Chartrand, 1999; Bargh et al., 2001; Custers and Aarts, 2005), while interpersonal, instrumental, and malicious motives imply heightened awareness (Schooler and Schreiber, 2005; Touré-Tillery and Fishbach, 2014).

Several studies have tried to classify the motives for deception using different methods, such as researchers’ expert judgment, literature reviews, and analysis of diary records and, interviews, or surveys (Turner et al., 1975; Ekman et al., 1989; DePaulo et al., 1996; Kashy and DePaulo, 1996; McLeod and Genereux, 2008; Phillips et al., 2011; Arcimowicz et al., 2015; Levine et al., 2016). However, few studies have designed self-report instruments to identify and categorize motives using factor analysis. One of the self-report instruments proposed, designed by Hart et al. (2019), identifies two categories that evaluate relational and antisocial motives. In a later study, Hart et al. (2020) found three categories of motives for lying: self-serving lies (such as avoiding the consequences of bad behavior and self-promotion), altruistic or benevolent lies (to benefit another), and vindictive lies to harm another person.

The aim of this study is to analyze the psychometric properties of an instrument that assesses people’s main motives for lying in their daily lives. The instrument was constructed to combine the different theoretical models described, as well as other typologies proposed by various authors on the motives for lying. The CEMA-A questionnaire was based on a review of the literature, to integrate the various motives behind every day lies. The instrument design mainly took into account the role of emotions in lying (Ekman, 1985/2001; Tangney et al., 1996); the five motivations for lying proposed by Turner et al. (1975); the three main reasons/motives of Buller and Burgoon (1996); the 10 pancultural deception motives of Levine et al. (2016); the research on self-presentational motives for lying in everyday life (DePaulo et al., 1996, 2003a; Kashy and DePaulo, 1996; DePaulo and Kashy, 1998), and personality variables related to lying (Olson and Weber, 2004; McLeod and Genereux, 2008; Armas-Vargas, 2017a; Armas-Vargas, 2020; Armas-Vargas, 2021b). In a pilot study (Armas-Vargas, 2021a), a four-factor structure was obtained, after exploratory factor analysis (EFA). The “Intrapersonal Motivation–Emotionality” category evaluates motives related to self-deception and negative emotions (shame, insecurity, fear of rejection and criticism). “Interpersonal Motivation–Sociability” evaluates reasons for the benefit of social relationships (to excuse or justify oneself, avoid conflicts with others, and for reasons of a prosocial nature). “Egoism/Hardness Motivation” measures motives related to using relationships for one’s own benefit (to obtain advantage, manipulate others, present a good image and impress others). And finally, the “Malicious Motivation” category evaluates motives related to covert or direct harm, or false accusations that cause harm (Armas-Vargas, 2021a). Unlike the test proposed by Hart et al. (2019), the CEMA-A posits two new categories: Intrapersonal Motivation and Egoism/Hardness Motivation. The other two factors of relational and antisocial motives proposed by Hart et al. (2019) correspond, to a certain extent, with Interpersonal Motivation–Sociability and Malicious Motivation, respectively.

The objective of this work is to study the psychometric properties of the CEMA-A instrument. Specifically, it will analyze whether the factorial structure found in the previous exploratory analyses (Armas-Vargas, 2021a), remains stable. Next, confirmatory factor analysis (CFA) will be used to check construct validity whether the data conform to the proposed four-factor structure. The internal consistency of the four scales and the total test will be studied, along with the temporal stability provided by the test–retest correlations of the factors. Likewise, factorial invariance will be examined to verify whether the structure is similar between men and women. Convergent and discriminant validity will be checked by analyzing the relationship with other personality variables. Finally, the mean differences of the various factors of the CEMA-A will be analyzed according to gender and level of education.

## 2 Materials and methods

### 2.1 Participants

The total sample was 1,722 adults (Sample 3) from the general population of the Canary Islands (Spain), aged 18 to 77 years (Mage = 35.13, SD = 13.74): 55.89% women (*N* = 962) and 44.11% men (*N* = 760). The total sample was divided into subsamples for the different phases of the study. Sample 1 consisted of 520 participants aged 18 to 76 years (Mage = 36.80, SD = 14.44) and was used to perform the EFA. Sample 2 consisted of 1,202 participants aged 18 to 77 years (Mage = 34.41, SD = 13.37), and was used for CFA and analysis of invariance, based on gender. Sample 3 was used to perform analysis of variance (MANOVA) based on gender and level of education. Sample 4 consisted of 529 participants from the total sample, aged 18 to 71 years (Mage = 34.90, SD = 13.25), selected to analyze the temporal stability of the factors. Table 1 presents the characteristics of the total sample and the different subsamples.

**Table 1:** Sociodemographic characteristics of the participants.

	Sample 1	Sample 2	Sample 3	Sample 4
	(n = 520)	(n = 1,202)	(n = 1722)	(n = 529)
Sex (Women, Men) (%)	(55.77/44.23)	(55.95/44.05)	(55.89/44.11)	(50.28/49.72)
Age (*M*, SD)	36.80 (14.44)	34.41 (13.37)	35.13 (13.74)	34.90 (13.25)
*Civil Status (%)*				
Single	65.31	68.55	67.57	63.33
Married	25.97	22.71	23.70	25.74
Separated	3.10	2.10	2.40	8.35
Divorced	5.62	6.64	6.33	2.58
*Level of education (%)*				
Primary	3.08	4.49	4.06	5.29
Secondary	14.23	13.81	13.94	16.24
Baccalaureate/Technical studies	38.65	44.43	42.69	55.78
University	44.04	37.27	39.31	22.69

### 2.2 Instruments

Questionnaire for the Evaluation of Deceit, Lies and Self-deception (CEMA) (Armas-Vargas, 2021a). The instrument was developed based on Muñiz and Fonseca-Pedrero (2019) recommendations for test construction. This self-report instrument designed to assess variables associated with “deceit, lying, concealment, and self-deception” consists of four sub-questionnaires: The Motives for Lying (CEMA-A); Opinions about Self-Deception Lying (CEMA-B); Content of Lies (CEMA-C); and Receivers of the Lies (CEMA-D). In this study, we validate the CEMA-A subquestionnaire that assesses people’s motives for lying in their daily lives. Questionnaire development drew from a pool of 80 items related to personal–emotional variables (associated with protection of the self, such as fear of rejection, fear of what others will say, insecurity, self-esteem problems, self-deception); items related to instrumental content, manipulation of others, pro-image, and self-presentation (more selfish, intention to benefit oneself); other items concerning lies in social interactions (lies that are altruistic, prosocial, or beneficial to others); and finally, items related to malice or harming others. Two independent experts checked the wording and clarity of the items; when they disagreed, a third expert was consulted. Participants were informed that the aim of the study was to investigate the motives people may have for lying. Specifically, participants received the information that “lying includes both deliberately omitting relevant information and telling someone something that is not true.” Then, to minimize problems of social desirability, participants were also told that it is normal to lie from time to time and the fact of being able to lie is not censored, but research is interested in studying the reasons why one might lie at some point. Finally, participants were asked to indicate the reasons or motives for which they usually deceive, lie, or withhold information from others and to indicate on a Likert-type scale of seven alternatives (1 = rarely, 2 = from time to time, 3 = sometimes, 4 = usually, 5 = very often, 6 = many times, and 7 = always), which of the listed motives they generally use to a greater or lesser extent. They were thanked for their participation and asked to be honest in their answers.

In the previous pilot study (Armas-Vargas, 2021a), an exploratory factor analysis (oblimin rotation) was applied. Items that saturated on two factors and items with factor loadings below 0.40 were eliminated from the factor analysis, reducing the number of items from 80 to 45. The CEMA-A questionnaire was finally composed of 45 items, and a factorial structure of four factors or general categories was obtained: Intrapersonal Motivation–Emotionality, Interpersonal Motivation–Sociability, Egoism/Hardness Motivation, and Malicious Motivation. The Intrapersonal Motivation–Emotionality category evaluates motives related to self-deception and negative emotions; Interpersonal Motivation–Sociability collects motives related to maintaining positive social relationships; Egoism/Hardness Motivation measures motives related to using relationships for one’s own benefit; and the Malicious Motivation category evaluates motives related to covert or direct harm, or false accusations that cause harm (Armas-Vargas, 2021a). Interpersonal Motivation–Sociability and Egoism/Hardness Motivation both refer to the domain of interpersonal relationships. However, in the Egoism/Hardness motives, the intention of the individual who lies is to benefit him/herself with the act of lying, whereas in the Interpersonal Motivation–Sociability, the intention of the individual is more prosocial: he/she intends to benefit others with the act of lying. On the other hand, the Intrapersonal Motivation–Emotionality factor is related to more personal motivations, where the person “avoids or does not want to face the truth and reality,” indirectly obtaining a “self-benefit, without instrumentalizing anyone” by avoiding facing reality. In the Egoism/Hardness Motivation factor, the person intends to gain self-benefit by “manipulating and instrumentalizing others.” In this second case, the person acts and confronts reality in order to achieve a certain goal. The total reliability of Cronbach’s alpha was 0.97 and the omega coefficient ωj = 0.79.

Eysenck Personality Questionnaire − Revised (EPQ-R; Eysenck and Eysenck, 1997). It explores three personality traits: (1) Extraversion (sociable, active, assertive, sensation-seeking); (2) Neuroticism (anxious, depressed, guilt); and (3) Psychoticism (aggressive, cold, egocentric, impulsive, antisocial). It also includes the Lie scale, intended to measure the tendencies of examinees to “fake good” when they complete the questionnaire. It is made up of 83 items with two response alternatives (true or false), referring to the person’s way of acting, feeling and thinking. Because it is a shorter tool, the EPQ-R was used in this study to assess the personality characteristics that have been linked to lying, such as psychoticism, neuroticism, and extraversion. Since no other tests of motives for lying have been validated in Spanish, the EPQ-R was used to assess convergent validity through the lie scale, along with discriminant validity, to distinguish between motives for lying and personality traits that have previously been weakly correlated (Gudjonsson and Sigurdsson, 2004; McLeod and Genereux, 2008; Hart et al., 2019). Internal consistency oscillates between 0.71 and 0.86.

### 2.3 Procedure

Data collection was done by fourth-year psychology undergraduates and master’s students of general health psychology at the University of La Laguna for three academic years 2020–2023. This study was not preregistered. Samples 1 (*N* = 520) and 2 (*N* = 1,202) were obtained in 2020, and 2021 and 2023, respectively. Sample 3 (*N* = 1722) is the sum of both samples, and Sample 4 (*N* = 529) was randomly drawn from the whole sample. The students were trained to administer the aforementioned tests, order to play the role of evaluators. Sampling was incidental for convenience (Gil-Escudero and Martínez-Arias, 2001). The students had to select 15 to 20 people from their close environment, homogenized by gender, to whom they would apply the instrument. They were informed about the objective of the study, voluntarily accepted to collaborate, and gave their written informed consent. Participants received an envelope containing an identification code and tests. One week later, the sealed envelope was collected, to guarantee anonymity. Participants were instructed to write a contact telephone number on the envelope, so that they could be contacted for a second retest. After four weeks, half of the sample of 1,200 was randomly selected and, of the 600 participants selected, 529 had returned the envelope with the retest completed. The participants completed the questionnaires independently, at home and on paper in approximately 30 min. No reward was offered for participation. The study was carried out in accordance with the Declaration of Helsinki and was approved by the Research Ethics and Animal Welfare Committee of the University of La Laguna (Registration Number: CEIBA2023-3299).

### 2.4 Data analysis

The data were analyzed using R version 4.0.5 (R Core Team, 2017), the Lavaan package (Rosseel, 2012), and the syntax described by ULLRToolbox (Hernández and Betancort, 2018). Initially, an EFA was performed with Sample 1 (*N* = 520). This sample was used to verify whether the same four-factor structure remained stable with 45 items proposed by Armas-Vargas (2021a). The procedure used to determine the number of factors was the optimal application of Horn’s parallel analysis (Timmerman and Lorenzo-Seva, 2011). An EFA was performed on principal axes and oblique rotation (oblimin) since a correlation between the factors was expected.

Secondly, CFA was performed with 1,202 participants (Sample 2). The objective was to check the factorial structure of the questionnaire using the four-factor model obtained previously. The model fit was estimated using the maximum likelihood estimation method (Brown, 2006) was verified using a comparative fit index (CFI), Tucker-Lewis Index (TLI), root mean square error of approximation (RMSEA) and standardized root mean square residual (SRMR), reported in the bibliography as adequate for ordinal data (Abad et al., 2011; Byrne, 2012). The expected values for an acceptable fit were around 0.90 for the CFI, TLI, normed fit (NFI) and non-normed fit (NNFI) indices (Kline, 2011). Values under 0.05 for SRMR and under 0.10 for RMSEA, with a 90% confidence interval, indicate reasonable model fit (Browne and Cudeck, 1989; MacCallum et al., 1996). To statistically compare the four-dimensional model, we used the *χ*^2^ difference test. The reliability of the CEMA-A was evaluated using omega coefficient (McDonald, 1999). The omega coefficient (ω) is more precise than Cronbach’s alpha (α) because reliability can be directly calculated using the estimates of the CFA parameters, resulting in much greater stability when dealing with non-continuous data (Gadermann et al., 2012; Dunn et al., 2014).

Thirdly, the factorial invariance of the CEMA-A based on gender was analyzed with the 1,202 participants (Sample 2), using the multigroup CFA. The configural invariance test shows whether the same items are associated with the same construct. After checking the configural invariance we tested the metric invariance by restricting the factorial loadings of similar items, so that they were the same in the different groups. To determine the metric invariance of the groups, we performed a Δ*χ*^2^ test (Sass, 2011). If the metric model does not differ from the configural model, the metric invariance is inferred.

Fourthly, to analyze convergent and discriminant validity, Sample 1 participants completed the EPQ-R questionnaire. The association between the CEMA-A and the EPQ-R scales was analyzed using Pearson correlation.

Fifthly, with 1,722 participants (Sample 3), we analyzed the mean differences of the different factors of the CEMA-A by MANOVA, according to gender and level of education. The MANOVA effect size was estimated using partial η2, considering 0.01 as small, 0.06 as medium and 0.14 as large.

Finally, we used the test–retest method (Aldridge et al., 2017) to analyze the stability of the CEMA-A (Sample 4), after four weeks. Vuong’s (1989) test was used to compare the predicted probabilities of non-nested models. First, it allows us to check whether two models are distinguishable, and then, to determines whether the second model shows a better fit than the first. Under the premise of the null hypothesis, it is proposed that the two non-nested models fit equally well, that is, the expected value of their log-likelihood coefficient is equal to zero.

## 3 Results

### 3.1 Exploratory factor analysis

**Figure 1:**
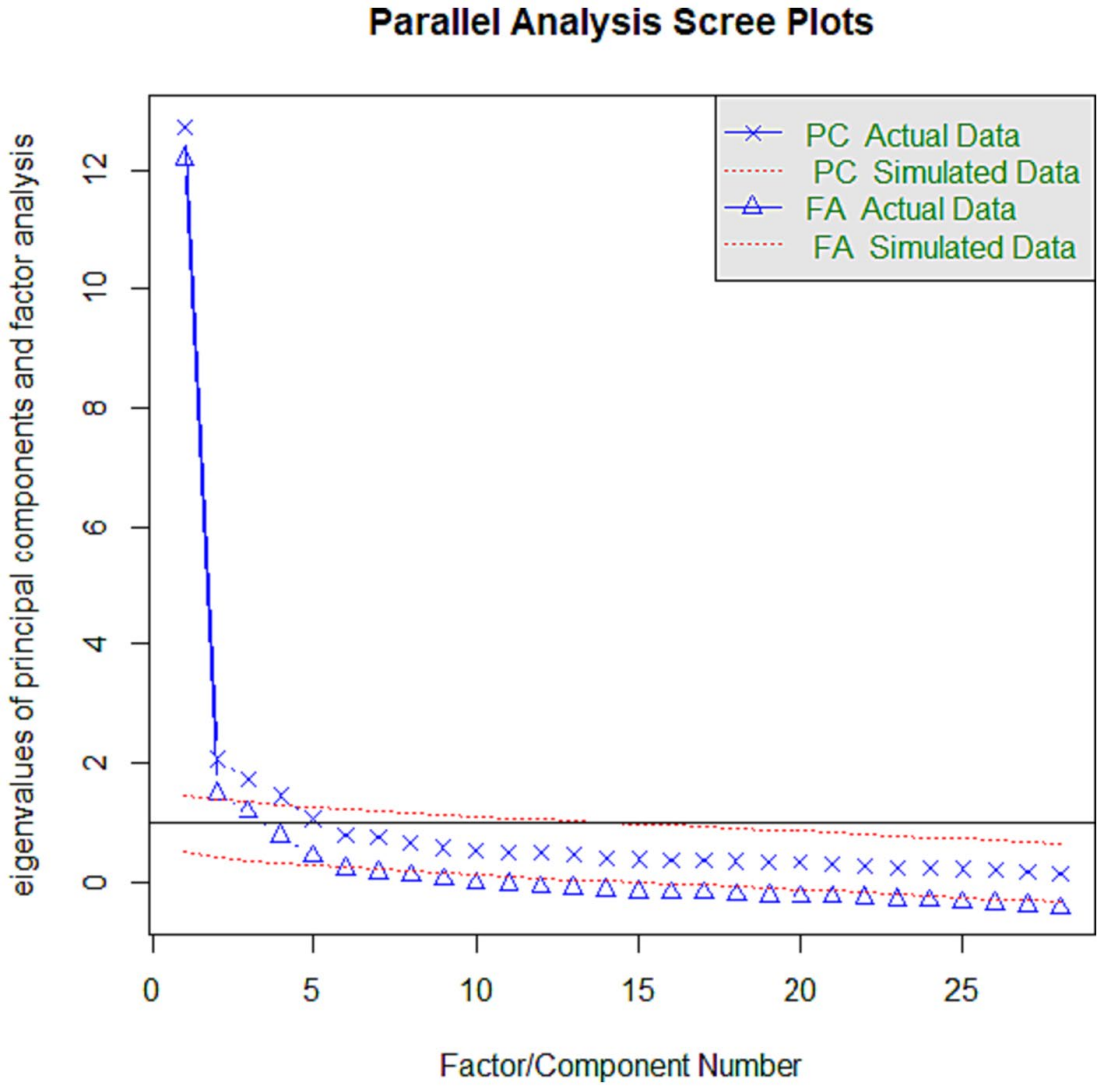
Parallel analysis scree plots.

Sample 1 (*N* = 529) was used to verify that the properties of the data were adequate to perform EFA. The Kaiser–Meyer–Olkin index (KMO = 0.96) and Bartlett’s test of sphericity were significant (*χ*^2^(990) = 16,501; *p* < 0.001), indicating that the analysis was feasible. The parallel analysis method (Horn, 1965) was used to decide the number of factors to extract. The scree test is a graphical representation of the magnitude of the eigenvalues and helps to identify the optimal number of factors that should be extracted. The scree test yielded only four factors that were included in the final scale (Figure 1). EFA (Sample 1) showed a four-factor structure with 43 items that explained 54.35% of the total variance (Intrapersonal Motivation, 18.37%; Egoism/Hardness Motivation, 15.45%; Interpersonal Motivation, 14.77%; and Malicious Motivation, 5.75%). Table 2 shows the standard deviation, skewness, kurtosis, and factor loading of each item. Of the 45 original CEMA-A items, two were deleted (item 23: “To feign a life I do not have”; item 32: “Out of jealousy”) because their means were too low and produced a floor effect. Table 3 shows the eigenvalue, explained and cumulative variance, as well as the Cronbach’s alpha and Omega hierarchical reliability of the four factors of the CEMA-A with the 43 items.

**Table 2:** Mean (*M*), standard deviation (*SD*), skewness, kurtosis and factor loading for CEMA-A (43 items).

Factor loading CEMA-A								
Reagents	*M*	*SD*	Skewness	Kurtosis	F1	F2	F3	F4
19. For fear of facing reality.	1.88	1.21	1.71	3.01	0.85			
17. Not to face the truth.	1.85	1.25	1.88	3.68	0.85			
10. Because I do not accept myself as I am.	1.65	1.14	2.11	4.60	0.78			
22. Because I feel insecure.	1.99	1.31	1.58	2.29	0.72			
16. Not to reveal my own meanness.	1.85	1.21	1.89	4.11	0.71			
30. Because it’s hard for me to accept things as they are.	1.71	1.10	1.85	3.51	0.69			
24. For fear of what they will say.	2.22	1.39	1.08	0.61	0.60			
38. Out of shame to admit the truth.	1.97	1.28	1.57	2.25	0.59			
13. To be accepted by others.	1.90	1.31	1.60	2.06	0.39			
39. Because “telling the truth” hurts more.	2.12	1.41	1.50	1.83	0.33			
7. Because it is easier for me to lie than tell the truth.	1.88	1.27	1.63	2.43	0.28			
9. Due to mistrust.	2.25	1.38	1.37	1.68	0.27			
29.To get an advantage over others.	1.59	1.03	2.28	6.08		0.91		
6. To try to win an argument with someone.	1.73	1.17	1.91	3.75		0.83		
44. Because it is easier to manipulate others.	1.51	1.03	2.70	8.28		0.79		
36. To benefit from something.	1.98	1.35	1.56	2.12	−0.26	0.76		
12. To get what I want.	2.04	1.35	1.55	2.04		0.68		
5. To impress others.	1.88	1.32	1.89	3.50		0.63		
42.To earn the respect and admiration of others.	1.63	1.19	2.34	5.68		0.62		
27. To give a better image of myself.	1.99	1.30	1.45	1.69		0.43		
28. To seek the approval of others.	1.58	1.02	2.13	4.89		0.38		
45. Because it helps me to relate.	1.66	1.12	2.07	4.41	0.27	0.38		
37. Because I cannot help it.	1.47	0.97	2.67	8.53		0.34		
18. To avoid problems with others.	2.78	1.42	0.91	0.55			0.77	
33. To avoid having to explain.	2.72	1.47	1.07	0.83			0.74	
35. To make others feel good.	2.76	1.55	0.91	0.22			0.71	
20.To hide certain information.	2.66	1.43	1.15	1.12			0.69	
2. So as not to offend others.	3.35	1.52	0.50	−0.44			0.62	
34. To hide something I know is wrong.	2.44	1.36	1.10	1.00			0.57	
43.To be kind and cordial to others.	2.51	1.40	0.96	0.55			0.54	
14. For fear of punishment.	2.27	1.33	1.26	1.68			0.47	
11. To hide certain problems or difficulties.	2.40	1.38	1.15	1.06	0.35		0.44	
15. To protect myself.	2.38	1.43	1.11	0.68	0.27		0.36	
21. To defend myself against the attacks of others.	2.10	1.34	1.30	1.18		0.29	0.30	
3. To save face.	2.56	1.49	0.87	0.21			0.29	
26.To avoid telling or acknowledging the truth.	1.94	1.20	1.77	3.77			0.28	
41.To avoid taking responsibility for something.	2.09	1.25	1.25	1.48			0.27	
25. To give a bad image of another person.	2.02	1.39	1.47	1.73			0.34	0.69
8. To give false information about another person.	1.69	1.22	2.12	4.43				0.59
4. Not to make others feel bad.	2.59	1.58	0.91	0.03			0.39	−0.58
40. To falsely accuse another person and cause them harm.	1.41	0.89	2.53	6.68	0.28			0.49
1. To raise doubts about another person.	1.88	1.37	1.65	2.03				0.49
31.To make the other feel guilty.	1.57	1.11	2.38	6.08				0.48

F1, Intrapersonal motivation; F2, Egoism/Hardness motivation; F3, Interpersonal motivation; F4, Malicious motivation.

**Table 3:** Factor analysis of the CEMA-A questionnaire (*N* = 520).

	Eigenvalue	Explained Variance (%)	Cumulative variance (%)	Proportion explained (%)	α	ωj
Intrapersonal motivation	7.90	18.37	18.37	33.80	0.93	0.86
Egoism/Hardness motivation	6.64	15.45	33.82	28.43	0.93	0.80
Interpersonal motivation	6.35	14.77	48.59	27.18	0.92	0.81
Malicious motivation	2.47	5.76	54.35	10.59	0.75	0.70

Total reliability α = 0.96 and ωj = 0.81.

Next, a first-order EFA was applied again. Items whose factor loading was <0.40 and those that saturated in two factors were eliminated (≥ 0.30). Based on these criteria, the following items were eliminated: 7, 9, 13 and 39 of the Intrapersonal Motivation factor; items 23, 28, 32, 37, and 45 of the Egoism/Hardness Motivation factor; items 3, 11, 15, 21, 26, and 41 of the Interpersonal Motivation factor; and items 4 and 25 of the Malicious Motivation factor. With the 28 items, the KMO index was 0.95 and Bartlett’s sphericity test was again significant (*χ*^2^(378) = 10,026; *p* < 0.001). The four-factor structure was maintained with the 28 items. Internal consistency was calculated using Cronbach’s alpha and Hierarchical Omega, which were 0.95 and 0.77, respectively, for the total scale. The Intrapersonal Motivation factor showed α = 0.92 and ωj = 0.72; the Egoism/Hardness Motivation factor, α = 0.93 and ωj = 0.83; the Interpersonal Motivation factor, α = 0.89 and ωj = 0.77; and for the Malicious Motivation factor it was α = 0.77 and ωj = 0.72. Of the final structure of 28 items, the factors for Intrapersonal Motivation, Egoism/Hardness, Interpersonal Motivation, and Malicious Motivation explained 18.74, 16.93, 15.84, and 6.59% of the total variance, respectively. As can be seen, the correlation between the different factors was high, mainly between Intrapersonal Motivation and Egoism/Hardness Motivation (*r* = 0.70) (Table 4).

**Table 4:** Correlations between CEMA-A factors (*N* = 520).

CEMA-A			
CEMA-A	Intrapersonal motivation	Egoism/Hardness motivation	Interpersonal motivation
Intrapersonal motivation	−		
Egoism/Hardness motivation	0.70***	−	
Interpersonal motivation	0.66***	0.62***	−
Malicious motivation	0.54***	0.56***	0.54***

*p < 0.05, **p < 0.01, ***p < 0.001.

### 3.2 Confirmatory factor analysis

To study the dimensional structure of the scale, we performed CFA with Sample 2, based on the model obtained with Sample 1. To analyze construct validity, we used a four-factor model with the 28 items, using the maximum likelihood estimation method. Figure 1 displays the results of the CFA of the four-factor model. To better evaluate the model parameters, taking into account the recommendations of other authors (Brown, 2015), we considered several indices simultaneously. Figure 1 shows the best fit model and normalized path coefficients for each variable observed. All item loadings were found to be at an acceptable level (≥ 0.47), and all parameter estimates were significantly different from 0. Latent correlation indices between model factors were high, for example, the latent correlation between the Egoism/Hardness and Malicious Motivation factors was r = 0.81.

**Figure 2:**
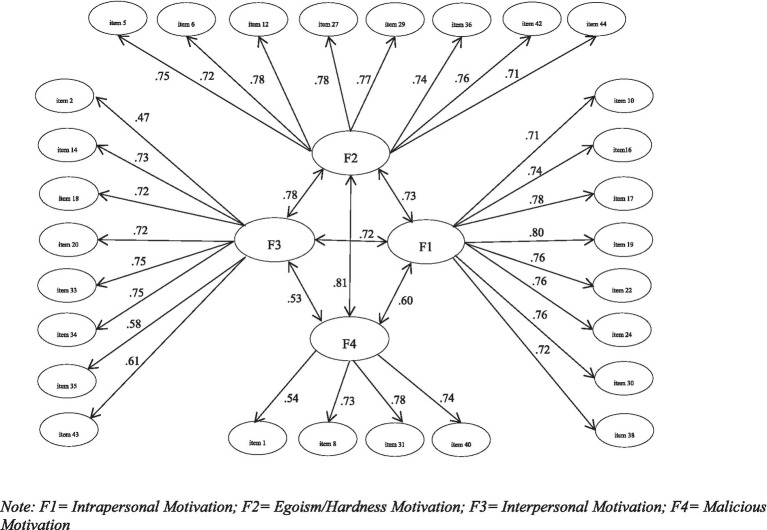
CEMA a four factor models. F1, Intrapersonal Motivation; F2, Egoism/Hardness Motivation; F3, Interpersonal Motivation; F4, Malicious motivation.

When the proposed theoretical model was tested (Figure 2), an adequate fit to the data was obtained (Table 5). Applying the good fit statistics in this model resulted in the following: (*χ*^2^ = 1,460.97, df = 325, *p* < 0.001; CFI = 0.94; TLI = 0.93; NFI = 0.93; NNFI = 0.93; RMSEA = 0.05, CI = 0.051–0.057; SRMR = 0.04). It should be noted that all the parameters indicated in Figure 2 (factorial loadings, correlation between factors and measurement errors of the items) were significant for *p* < 0.001. Internal consistency was calculated using the McDonald omega coefficient for four factors. The Intrapersonal motivation factor presented ω = 0.91, the Egoism/Hardness motivation factor, ω = 0.88, the Interpersonal motivation factor, ω = 0.84, and the Malicious motivation factor, ω = 0.79.

**Table 5:** Factor loading and internal consistency of latent variables.

Parameter estimate	Un-standard β	z	Standard Β	Ω McDonald
Intrapersonal motivation → item 10	1		0.71	0.91
Intrapersonal motivation → item 16	1.08	24.48***	0.74	
Intrapersonal motivation → item 17	1.17	25.88***	0.78	
Intrapersonal motivation → item 19	1.20	26.14***	0.80	
Intrapersonal motivation → item 22	1.26	28.25***	0.76	
Intrapersonal motivation → item 24	1.14	24.62***	0.76	
Intrapersonal motivation → item 30	1.09	25.15***	0.76	
Intrapersonal motivation → item 38	1.13	24.16***	0.72	
Egoism/Hardness motivation → item 5	1		0.75	0.88
Egoism/Hardness motivation → item 6	0.99	27.98***	0.72	
Egoism/Hardness motivation → item 12	1.11	27.29***	0.78	
Egoism/Hardness motivation → item 27	1.08	27.43***	0.78	
Egoism/Hardness motivation → item 29	0.86	26.88***	0.77	
Egoism/Hardness motivation → item 36	1.01	25.52***	0.74	
Egoism/Hardness motivation → item 42	0.88	26.59***	0.76	
Egoism/Hardness motivation → item 44	0.78	23.04***	0.71	
Interpersonal motivation → item 2	1		0.47	0.84
Interpersonal motivation → item 14	1.46	15.5***	0.73	
Interpersonal motivation → item 18	1.47	15.57***	0.72	
Interpersonal motivation → item 20	1.43	15.58***	0.72	
Interpersonal motivation → item 33	1.56	15.72***	0.75	
Interpersonal motivation → item 34	1.48	15.81***	0.75	
Interpersonal motivation → item 35	1.22	16.28***	0.58	
Interpersonal motivation → item 44	1.21	15.80***	0.61	
Malicious motivation → item 1	1		0.54	0.79
Malicious motivation → item 8	1.42	17.10***	0.73	
Malicious motivation → item 31	1.58	17.61***	0.78	
Malicious motivation → item 40	1.26	17.24***	0.74	

***p < 0.001.

### 3.3 Invariance of the CEMA-A factorial structure

Multigroup Confirmatory Factor Analysis. To check whether the factorial structure was similar according to gender (configural invariance), the parameters were estimated simultaneously for each gender level. The multigroup CFA fit indices were (*χ*^2^ = 2082.76, df = 650, *p* < 0.001; CFI = 0.93; TLI = 0.92; NFI = 0.90; NNFI = 0.92; RMSEA = 0.06, CI = 0.058–0.063; SRMR = 0.048). Therefore, we can conclude that both the number of factors and the factor loading pattern of the items on the CEMA-A scale are similar for men and women.

Regarding metric invariance, the fit indices were acceptable according to gender (*χ*^2^ = 2111.75, df = 674, *p* < 0.001; CFI = 0.93; TLI = 0.92; NFI = 0.90; NNFI = 0.92; RMSEA = 0.06, CI = 0.057–0.062; SRMR = 0.051). The results show that the fit indices between the configural model and the metric model did not differ according to gender (Δ*χ*^2^ = 28.99, Δdf = 24, *p* = 0.220) (see Table 5).

### 3.4 Differences in the sociodemographic data

To explore whether the CEMA-A questionnaire was useful for differentiating the motives for lying of people with different sociodemographic profiles, MANOVA was performed with the total sample (Sample 3). The Intrapersonal, Interpersonal, Egoism/Hardness, and Malicious Motivation scales were taken as dependent variables, and gender and educational level as independent variables. Significant differences were found according to gender [*F* (1,1718) = 21.04, *p* < 0.001]. Specifically, men scored higher than women in the Egoism/Hardness and Malicious Motivation scales (Table 6).

**Table 6:** Comparison of gender with CEMA-A Factors.

	Men		Women			
	(*N* = 760)		(*N* = 962)			
	*M*	*SD*	*M*	*SD*	*F*	η^2^
Intrapersonal motivation	15.41	8.26	15.34	7.96	0.03	0.00
Interpersonal motivation	21.80	8.91	21.07	8.46	3	0.00
Egoism/Hardness motivation	16.20	8.65	13.67	7.19	43.93***	0.03
Malicious motivation	6.13	3.14	5.44	2.80	22.82***	0.01

*p < 0.05, **p < 0.01, ***p < 0.001.

Regarding educational level, the MANOVA showed significant differences [*F* (3,1719) = 1.9, *p* < 0.05], particularly in the Interpersonal Motivation factor. However, after analyzing the *post-hoc* contrasts, no significant differences were found between the different levels of education (Table 7).

**Table 7:** Comparison of educational level with CEMA-A factors.

	Primary (*N* = 70)		Secondary (*N* = 240)		Baccalaureate (*N* = 736)		University (*N* = 677)			
	*M*	*SD*	*M*	*SD*	*M*	*SD*	*M*	*SD*	*F*	η^2^
Intrapersonal motivation	15.37	9.10	15.12	8.15	15.66	8.28	15.15	7.76	0.55	0.00
Interpersonal motivation	19.66	7.70	20.38	8.54	21.86	9.07	21.42	8.32	2.77*	0.00
Egoism/Hardness motivation	14.17	78.27	15.00	7.97	14.99	8.09	14.54	7.90	0.58	0.00
Malicious motivation	5.97	3.19	5.99	3.31	5.80	3.04	5.59	2.74	1.39	0.00

*p < 0.05, **p < 0.01, ***p < 0.001.

### 3.5 Convergent and discriminant validity

Convergent and discriminant validity was analyzed using Pearson’s correlation between the CEMA-A and EPQ-R scales (Sample 1). All the CEMA-A factors correlated positively with Neuroticism and Psychoticism, and negatively with L scale, suggesting convergent validity (Table 8). The highest correlations were between Neuroticism and Intrapersonal Motivation (*r* = 0.37; *p* <0.001), as well as between Psychoticism with Egoism/Hardness Motivation (*r* = 0.29; *p* < 0.001) and with Malicious Motivation (*r* = 0.31; *p* <0. 001). The Extraversion factor demonstrated discriminant validity, since no significant correlations were found with the CEMA-A factors, except for a low negative correlation with Intrapersonal Motivation (*r* = −0.09; *p* < 0.05).

**Table 8:** Correlations between CEMA-A factors and the EPQ-R personality questionnaire.

	EPQ-R			
CEMA-A	Extraversion	Neuroticism	Psychoticism	L scale
Intrapersonal motivation	−0.09*	0.37***	0.15***	−0.30***
Interpersonal motivation	−0.01	0.22***	0.11*	−0.31***
Egoism/Hardness motivation	0.05	0.21***	0.29***	−0.30***
Malicious motivation	−0.03	0.13***	0.31***	−0.18***

*p < 0.05, **p < 0.01, ***p < 0.001.

### 3.6 Score stability (test–retest)

Vuong’s (1989) test was applied to assess whether there were differences between the two non-nested models. Both models were verified as indistinguishable (variance test), and the fit of both models was equal for the focal population (non-nested likelihood ratio test) in the four categories of motivations to lie. The test–retest correlation was 0.86 for Intrapersonal Motivation (pretest: z 0.825, *p* = 0.21; retest: z 0.825, *p* = 0.80), 0.81 for Intrapersonal Motivation (pretest: z 1.248, *p* = 0.10; retest: z 1.258, *p* = 0.90), 0.93 for Egoism/Hardness Motivation (pretest: z 1.225, *p* = 0.11; retest: z 1.225, *p* = 0.89), and 0.77, for Malicious Motivation, (pretest: z 0.616, *p* = 0.27; retest: z 0.616, *p* = 0.73).

## 4 Discussion

The aim of this study was to verify the stability of the factorial structure of the CEMA-A questionnaire in the Spanish adult population. The results showed that the CEMA-A has adequate psychometric properties and is valid and reliable instrument to measure different motives behind every day lies. The new structure of the of the 28-item CEMA-A instrument was confirmed, through EFA and CFA, and the four-factor model containing the factors Intrapersonal Motivation, Interpersonal Motivation, Egoism/Hardness Motivation and Malicious Motivation, which concurs with the factorial structure of the preliminary study of 45 items (Armas-Vargas, 2021a). Moreover, the temporal stability of the measurement instrument scores was verified.

The general category Egoism–Hardness Motivation of the CEMA-A encompasses various subcategories of motives focused on obtaining personal benefits, such as instrumental motives (item 12 “to get what I want”; item 36 “to benefit from something”), motives related to manipulation of others (item 44 “because it is easier to manipulate others”; item 6 “to try to win in an argument with someone”), or motives related to showing a positive self-image (item 5 “to impress others”; item 27 “to give a good image of myself”). Instrumental and manipulative motives are related to those proposed in Levine et al.’s (2016) pancultural model: “non-monetary personal advantage,” while the motives related to showing a positive self-image of the CEMA-A are equivalent, in a way, to Levine et al.’s (2016) “self-image management.” In the case of CEMA, it also includes the search for admiration. The general category Malicious Motivation of the CEMA-A includes content related to harming others (item 1 “to generate doubts about another person”; item 40 “to falsely accuse someone and cause harm”) and has a certain similarity with the “malicious” category of Levine et al.’s (2016) pancultural model. The contents of the Egoism/Hardness and Malicious Motivations find a parallel with the type of serious lies proposed by DePaulo et al. (2004). According to these authors, people who tell serious lies try to profit from dubious deals, and lie instrumentally to get what they want, and to avoid punishment. The truths behind serious lies are often shameful, immoral, or illegal (DePaulo et al., 2004; Palena et al., 2021). Similarly, people high on Machiavellianism tend to engage in “immoral” behaviors to achieve their goals (Monaghan et al., 2020).

The general category Interpersonal Motivation focuses on motives that try to maintain positive social relationships and includes content on prosocial-empathy (item 35 “to make others feel good”; item 43 “to be kind and cordial with others”), sociability and agreeableness (Item 2 “to not offend others”), hide information that could cause harm (item 11 “to hide certain problems or difficulties”), or avoid problems with others (item 18 “to avoid problems with others”; item 34 “to hide something that I know is wrong”). The content of this category is related to the motives proposed in Levine et al.’s (2016) pancultural model, such as altruistic lies, social politeness, personal transgression, and evasion, respectively.

The general category Intrapersonal Motivation includes new content related to self-deception that has not been addressed in the area of motives for lying in the literature (Armas-Vargas, 2021a). Some of the reasons related to self-deception are “so as not to face the truth” (item 17), “for fear of facing reality” (item 19), “because it is difficult for me to accept things as they are” (item 30), where self-deception occurs through denial of a real problem and acting as if it did not exist (Goleman, 1985; Cohen, 2001; Zerubavel, 2006; Friedrichs, 2014). At some point in their lives, people may be exposed to unpleasant or traumatic situations that lead to the need for self-deception in order to survive the negative experience. Self-deception is the result of a functional and adaptive system in the protection of the self and the regulation of goals. It is not pathological in itself, since most people use it at some point in their lives (Sirvent et al., 2019). Some authors consider that self-deception can lead to a gain, such as improving self-image (Starek and Keating, 1991; Bachkirova, 2016). Other authors emphasize its function as an avoidance strategy, such as avoiding distress (Fingarette, 1969; Sackeim, 1983). It has also been proposed that self-deception may arise from selective attention, whereby certain information is ignored or dismissed, despite evidence (Greenwald, 1997; Sharot, 2011), Other research suggests that self-deception is a cognitive process of biasing information to obtain or maintain a false belief that may be beneficial or detrimental to oneself (Mei et al., 2022). A close relationship has been found between self-deception and deception of others (Lu and Chang, 2014). Self-deception functions as an automatic mechanism of protection and adaptation of the “I,” which ultimately seeks to safeguard the psychic order (Armas-Vargas, 2020). These types of reasons fulfil the objective of hiding and/or denying evidence that we do not know or do not want to accept, which, if rejected, would leave us psychologically unprotected (Armas-Vargas, 2020, 2021a). Specifically, there is gain in self-deception: distress is avoided, real damage is minimized, and benefits such as subjective and interpersonal well-being and improving self-image are obtained (Friedrichs, 2014; Bachkirova, 2016). In the “process” of self-deception, many strategies that people use escape their control and awareness. Many implicit and automatic processes may be outside volitional reach (Bargh, 1990; Bargh et al., 2001). The evaluation of self-deception is therefore carried out as an experience already lived and past, whereby the person realizes (either by themselves or with the help of a professional) that they have been self-deceived (Armas-Vargas, 2017a,b, 2020). What is evaluated, therefore, is not the self-deception in the moment, but rather that the person was self-deceived (Martínez-Manrique, 2007).

In addition, intra-personally motivated lying includes personal and emotional reasons that evaluate content related to insecurity, problems of self-esteem, shame, or fear of what others will say (item 10 “because I do not accept myself as I am”; item 16 “so as not to reveal my own meanness”; item 22 “because I feel insecure”). These motives are responsible for adapting reality to our emotional and psychological needs, to protect our identity, self-esteem, and the image others have of us (Turner et al., 1975; Buller and Burgoon, 1996; Armas-Vargas, 2020, 2021a). Many of these emotional motives may be implicit or escape awareness (McClelland et al., 1989; Bargh and Chartrand, 1999; Bargh et al., 2001; Custers and Aarts, 2005).

The relationship between the CEMA-A and EPQ-R factors confirms convergent validity and evidences the role of personality in the motives for lying (Buller and Burgoon, 1996; Olson and Weber, 2004; McLeod and Genereux, 2008; Harhoff et al., 2023). One study found that coldness when lying (e.g., “I do not usually have remorse when I lie”) was positively related to Psychoticism, whereas emotional self-regulation when lying (e.g., “I feel guilty when I’m caught in a lie”) was negatively related. On the other hand, the Neuroticism factor has been found to positively correlate with Self-Deception, Insecurity, or Fear of Rejection and Criticism (Armas-Vargas, 2021b). Neuroticism has been related to the propensity to lie and to different types of lying (Phillips et al., 2011; Hart et al., 2020). Extraversion was not related to any of the CEMA-A factors, only showing a low negative correlation with Intrapersonal Motivation. Extraverted people tend to minimize, hide, and/or deny negative characteristics about themselves, to create a favorable impression to others (DePaulo et al., 1996; Tyler and Feldman, 2004; Armas-Vargas, 2021b).

Likewise, invariance analyses confirmed the equivalence for men and women of the measurements obtained by the instrument. Men scored higher in Egoism/Hardness Motivation and Malicious Motivation, which coincides with the pilot study (Armas-Vargas, 2021a). However, these differences must be taken with caution due to the small effect size found. However, Tyler and Feldman (2004) suggest that men and women may have different reasons for lying depending on circumstance. For women, lies are related to feigning positive feelings others, rather than being selfish (DePaulo et al., 1996; Tyler et al., 2006). A more self-centered lie may attempt to obtain a psychical rather than a monetary reward (DePaulo et al., 1996). These types of results can be explained through emotional variables, since women, tend to feel more distressed and see serious lies as less justifiable (DePaulo et al., 2004). Men tell more lies for their own benefit, despite potential harm to others, and more lies containing false information to manipulate others’ impressions of them (Phillips et al., 2011).

The CEMA-A has shown adequate psychometric properties, although certain limitations should be considered. Firstly, there is no consensus around a single type of motive for lying (Seiter and Bruschke, 2007; Guthrie and Kunkel, 2013). Secondly, the four categories do not include all the reasons for lying, they are not exhaustive or exclusive. Although the CEMA-A was constructed by sampling the different motives for lying that appear in the literature, as well as collecting those such as self-deception that were not assessed through self-report, future research may find other reasons not identified thus far. Thirdly, response biases may occur, both due to the content of the test itself (lies) and because it is a self-reported measure. This type of bias could be minimized by using a social desirability scale.

In future, analysis of the invariance in clinical and forensic samples, and in other cultures, could be interesting. Lying depends largely on the ethical and moral values of individuals and cultural conventions. Behaviors that are immoral in one culture may not be immoral in another (Kwiatkowska, 2015). Thus, it is important to identify whether the reasons for lying are similar, regardless of cross-cultural differences. Conversely, the reasons may vary, depending on whether the culture is individualistic or collectivist (Giles et al., 2019). In this sense, it could be of interest to adapt the CEMA-A to other cultures and verify its factorial invariance in different cultures. In addition, the CEMA-A questionnaire on motives for lying can be used to identify profiles of individuals according to their personality characteristics (e.g., the characteristics that define the person whose main motivation for lying is personal–emotional (fears, insecurity), as opposed to another whose motives are more focused on manipulating or instrumentalizing others). Previous research has shown that people with high anxiety, low self-esteem, and high Machiavellianism have motivations that will benefit them or others, whereas lies with protective motivation are associated with high empathy and low Machiavellianism (Cantarero et al., 2018). Furthermore, the CEMA-A could capture the motives for lying of different pathological populations, such as in the dark triad (psychopathy, Machiavellianism, and narcissism), where more malicious motives could appear. Michels et al. (2020) found a relationship between the dark triad and lying ability to achieve one’s objectives, though this relationship was moderated by intelligence. In the same line, it could be of interest to use an instrument on lies in the forensic population, such as gender violence, or in contentious procedures for the custody of children. Intrapersonal motives may appear in victims of gender violence, while in aggressors the motivation would be more instrumental or malicious. In men convicted of gender violence, self-deception and an absolutist morality have been found to explain in some way the violent behavior against their partners (Vecina, 2018). Future studies could examine whether the CEMA-A questionnaire is useful for identifying populations that have a greater propensity to lie, depending on type of motive.

In summary, the CEMA-A questionnaire is based on an exhaustive review of the literature on motives for lying, including from social psychology models and personality psychology. The instrument therefore provides an empirical framework to identify the various motives for lying. They are grouped into four broad categories in which intrapersonal motivation related to self-deception and individual differences, previously little studied as motives for lying in the literature, play a major role. The CEMA-A has proven to be an adequate instrument for identifying categories, motives, situations, and moments that lead to lying; it is the first instrument in Spanish to assess motives for lying. These findings have important practical implications and could be a useful tool for analyzing the motives for lying in different clinical, forensic, and/or employment contexts. These types of lies may be interesting for future research on lying and understanding liars.

## Data availability statement

The raw data supporting the conclusions of this article will be made available by the authors, without undue reservation.

## Ethics statement

The studies involving humans were approved by the Research Ethics and Animal Welfare Committee of the University of La Laguna (Registration Number: CEIBA2023-3299). The studies were conducted in accordance with the local legislation and institutional requirements. The participants provided their written informed consent to participate in this study.

## Author contributions

EA-V: Conceptualization, Data curation, Formal analysis, Investigation, Methodology, Project administration, Supervision, Validation, Visualization, Writing – original draft, Writing – review & editing. RM: Project administration, Supervision, Validation, Visualization, Writing – review & editing. JH-C: Data curation, Formal analysis, Methodology, Project administration, Software, Supervision, Validation, Visualization, Writing – review & editing.
