# Supplementary material for: Psychometric properties of the CEMA-A questionnaire: motives for lying
Source: Front Psychol. 2023 Dec 20;14:1289209. doi: 10.3389/fpsyg.2023.1289209 (PMC10765548; doi:10.3389/fpsyg.2023.1289209)
Supplement: Supplementary file 1 [file Table_1.DOCX]

Annex. Spanish version of the CEMA-A questionnaire **(Armas-Vargas, 2021)**

Instructions

Below are a number of phrases that relate to the reasons why people may lie or mislead others at some point during the day. Lying includes both leaving out relevant information on purpose and telling someone something that is not true. Lying happens often, and it is normal to do it from time to time.

Please read each sentence carefully and explain the reasons why you usually cheat, lie, or hide from others by ticking the alternative that best reflects your situation.

1 = rarely, 2 = from time to time, 3 = sometimes, 4 = usually, 5 = very often, 6 = many times and 7 = always

Please do not leave any item unanswered, and be honest in your answers.

Thank you for your participation.

What are the motives or reasons why you tend to deceive, lie, hide from others?

|  | rarely | from time  to time | sometimes | usually | very often | many times | always |
| --- | --- | --- | --- | --- | --- | --- | --- |
| 1. For fear of facing reality. | 1 | 2 | 3 | 4 | 5 | 6 | 7 |
| 2.To get an advantage over others. | 1 | 2 | 3 | 4 | 5 | 6 | 7 |
| 3. To avoid problems with others. | 1 | 2 | 3 | 4 | 5 | 6 | 7 |
| 4. To give false information about another person. | 1 | 2 | 3 | 4 | 5 | 6 | 7 |
| 5. Not to face the truth. | 1 | 2 | 3 | 4 | 5 | 6 | 7 |
| 6. Because it is easier to manipulate others. | 1 | 2 | 3 | 4 | 5 | 6 | 7 |
| 7. To avoid having to explain. | 1 | 2 | 3 | 4 | 5 | 6 | 7 |
| 8. Because I don´t accept myself as I am. | 1 | 2 | 3 | 4 | 5 | 6 | 7 |
| 9. To try to win an argument with someone. | 1 | 2 | 3 | 4 | 5 | 6 | 7 |
| 10. To make others feel good. | 1 | 2 | 3 | 4 | 5 | 6 | 7 |
| 11. To falsely accuse another person and cause them harm. | 1 | 2 | 3 | 4 | 5 | 6 | 7 |
| 12. Because I feel insecure. | 1 | 2 | 3 | 4 | 5 | 6 | 7 |
| 13. To benefit from something. | 1 | 2 | 3 | 4 | 5 | 6 | 7 |
| 14.To hide certain information. | 1 | 2 | 3 | 4 | 5 | 6 | 7 |
| 15. Not to reveal my own meanness. | 1 | 2 | 3 | 4 | 5 | 6 | 7 |
| 16. To get what I want. | 1 | 2 | 3 | 4 | 5 | 6 | 7 |
| 17. So as not to offend others. | 1 | 2 | 3 | 4 | 5 | 6 | 7 |
| 18. To raise doubts about another person. | 1 | 2 | 3 | 4 | 5 | 6 | 7 |
| 19. Because it´s hard for me to accept things as they are. | 1 | 2 | 3 | 4 | 5 | 6 | 7 |
| 20. To impress others. | 1 | 2 | 3 | 4 | 5 | 6 | 7 |
| 21. To hide something I know is wrong. | 1 | 2 | 3 | 4 | 5 | 6 | 7 |
| 22. For fear of what they will say. | 1 | 2 | 3 | 4 | 5 | 6 | 7 |
| 23.To earn the respect and admiration of others. | 1 | 2 | 3 | 4 | 5 | 6 | 7 |
| 24.To be kind and cordial to others. | 1 | 2 | 3 | 4 | 5 | 6 | 7 |
| 25.To make the other feel guilty. | 1 | 2 | 3 | 4 | 5 | 6 | 7 |
| 26. Out of shame to admit the truth. | 1 | 2 | 3 | 4 | 5 | 6 | 7 |
| 27. To give a better image of myself. | 1 | 2 | 3 | 4 | 5 | 6 | 7 |
| 28. For fear of punishment. | 1 | 2 | 3 | 4 | 5 | 6 | 7 |

Note**:**

Intrapersonal Motivation = 1, 5, 8, 12, 15, 19, 22, 26.

Egoism/Hardness Motivation = 2, 6, 9, 13, 16, 20, 23, 27.

Interpersonal Motivation = 3, 7, 10, 14, 17, 21, 24, 28.

Malicious Motivation = 4, 11, 18, 25.
